# Maslinic acid activates renal AMPK/SIRT1 signaling pathway and protects against diabetic nephropathy in mice

**DOI:** 10.1186/s12902-022-00935-6

**Published:** 2022-01-18

**Authors:** Huijuan Gao, Hong Wu

**Affiliations:** 1 Department of endocrinology, Daqing Oilfield General Hospital, No. 9 Zhongkang Street, Daqing, 163001 Heilongjiang Province China; 2 Department of endocrinology, Daqing Longnan Hospital, Aiguo Road No. 35, Daqing, 163453 Heilongjiang Province China

**Keywords:** Maslinic acid, Diabetic nephropathy, AMPK/SIRT1 signaling, Glomerulus

## Abstract

**Background:**

Diabetic nephropathy has been a devastating complication. Clinically, there is an urgent need for nephroprotective agents to delay the onset of diabetic nephropathy and ameliorate its symptoms. Maslinic acid is a pentacyclic triterpene acid with protective effect on multiple organs against oxidative stress and inflammation. In this research, we hypothesized that maslinic acid protects renal function against diabetic nephropathy.

**Methods:**

C57BL/6 J male mice administrated with 50 mg/kg of Streptozocin (STZ) daily were used to establish diabetic mouse model (blood glucose levels > 300 mg/dL). Urinary levels of albumin, total proteins, and creatinine were analyzed by an automatic analyzer. H&E staining was used to evaluate renal damage. qRT-PCR and ELISA were performed to investigate the inflammation and oxidative stress in renal tissues. Western blot was used to assess the activation of AMPK signaling.

**Results:**

Maslinic acid treatment alleviated the loss of body weight and blood glucose in diabetic mice. Renal structure and function were protected by maslinic acid in diabetic mice. 20 mg/kg maslinic acid treatment for 8 weeks significantly alleviated the oxidative stress and inflammation in the kidney of diabetic rats. Maslinic acid treatment activated the renal AMPK/SIRT1 signaling pathway.

**Conclusion:**

Maslinic acid ameliorates diabetic nephropathy and activates the renal AMPK/SIRT1 signaling pathway.

**Supplementary Information:**

The online version contains supplementary material available at 10.1186/s12902-022-00935-6.

## Introduction

Diabetic nephropathy is a major chronic microvascular complication of diabetes [1, 2]. Chronic high blood glucose level and systemic metabolic abnormality caused by diabetes lead to glomerulosclerosis and kidney damage [3]. Therefore, diabetic nephropathy is a component of diabetic systemic microangiopathy [4]. Diabetic nephropathy has been a leading cause of chronic kidney disease and end-stage renal disease (ESRD) [5]. At present, approximately 45% of ESRD patients in the United States suffer from diabetic nephropathy [6]. Diabetic nephropathy increases the morbidity and mortality of cardiovascular diseases in diabetic patients [7, 8]. Therefore, it is urgent to identify new agents to alleviate the symptoms of diabetic nephropathy and protect the renal function of diabetes patients.

Maslinic acid is a pentacyclic triterpene acid and a major bioactive component of Chinese traditional herbs including hawthorn, olive and red dates [9, 10]. The pharmacological properties of maslinic acid, including anti-cancer, anti-oxidation, anti-virus, anti-bacterial and anti-cancer activities, have attracted great attention from the medical community [11–13]. It has also been reported that maslinic acid can inhibit the apoptosis and necrosis of renal cells, protect the structure and function of the glomerulus, and thus have a therapeutic effect against the nephrotoxicity caused by cisplatin in rat models [14, 15]. In addition, accumulating evidence has demonstrated that maslinic acid alleviates the symptoms of diabetes and reduces the damage of the heart and liver caused by diabetes [16, 17]. However, the protective effect of maslinic acid in diabetic nephropathy and the underlying molecular mechanism remain unclear.

Adenosine 5′-monophosphate (AMP)-activated protein kinase (AMPK) is an important cell energy sensor, which is highly expressed in mesangial cells, glomerular endothelial cells and podocytes [18]. AMPK induces the activation of the insulin receptor tyrosine kinase, which in turn leads to the phosphorylation of its downstream substrate proteins. Therefore, AMPK is a positive regulator of insulin sensitivity, which plays a crucial role in regulating glucose uptake, enhancing insulin sensitivity, increasing fatty acid oxidation and regulating gene transcription [19]. Previous research has demonstrated that AMPK specific activation could reduce podocyte damage, maintain the integrity of the glomerular filtration barrier and delay the occurrence of diabetic nephropathy [18].

In this research, we demonstrated that 20 mg/kg maslinic acid treatment for 8 weeks repressed the oxidative stress and inflammation in the kidney of diabetic mice. We believed that our research could provide new evidence for the clinical applications of maslinic acid in treating diabetic nephropathy.

## Methods

### Animals

Eight-week-old C57BL/6 J male mice weighing between 18 to 22 g were obtained from Beijing Charles River Co., Ltd. (Beijing, China). All mice used in this research were cultured in the virus/antigen-free system with constant humidity and temperature. They were free to get the pathogen-free food and water. Streptozocin (STZ, Sigma Aldrich, MO, USA) was used to induce diabetic mouse model. A 0.1 mol/L sodium citrate solution was used to prepare a 10 mg/ml STZ solution. Fresh STZ solution was prepared in a dark room and filtered with a 0.22 μM filter. 50 mg/kg of STZ was injected intraperitoneally into each mouse on a daily basis for 5 days to induce diabetes, and the same amount of sodium citrate solution was used as negative vehicle control. Fasting blood glucose levels > 300 mg/dL was regarded as the incidence of diabetes. Animal studies were approved by Daqing Oilfield General Hospital.

### Experiment design

Diabetic mice of similar body weight were randomly divided into four groups: STZ (diabetic control group), MA5 (diabetic mice treated with 5 mg/kg maslinic acid), MA10 (diabetic mice treated with 10 mg/kg maslinic acid), and MA20 (diabetic mice treated with 20 mg/kg maslinic acid). Normal mice were in the normal control group (NC). Maslinic acid (MedChemExpress, Beijing, China) was injected intraperitoneally every 3 days after diabetes induction for 8 weeks. The changes in body weight and fasting glucose levels of mice were evaluated, respectively.

### Serum, urine, and renal tissue collection

The mice were kept in special metabolic cages (LaiAT technology co., LTD, Beijing, China) at the end of maslinic acid treatment for 24 h to collect their urine. Their urine volume was measured immediately and all urine samples were kept at − 20 °C for further analysis. Next, mice after 4 h-fasting were anesthetized with 50 mg/kg pentobarbital sodium. The blood of each mouse was collected by the capillary needle through their eyeball. Blood samples were centrifuged at 5000 rpm for 10 min at room temperature to collect serum. All serum samples were frozen at − 20 °C for further analysis. Finally, mice were sacrificed by cervical dislocation method, and their kidney were removed and kept on ice. Their renal tissues were cut in small pieces and stored at − 80 °C [20].

### Serum and urine parameters

Serum parameters including fasting blood glucose, blood urea nitrogen (BUN), urea, creatinine, and urine parameters including urine albumin, urine total protein, urine creatinine were analyzed by an automatic analyzer (Cobas® 8000 modular analyzer series. Roche Diagnostics, Berne, Switzerland). The analysis and calculation were previously demonstrated by Ali A. Shati and Zhang. et. Al [17], and the measurements were conducted for *n* = 14 per group [21].

### H&E staining

The renal tissue block of each mouse was fixed with a pre-prepared fixative (10% formalin). The tissue blocks were put into the embedding cassette, and the fixative fluid in the tissue was removed by washing with running water for 30 min. The tissues were dehydrated with increasing concentrations of alcohol, and xylene was used to replace the alcohol in the tissues. The tissue blocks were placed in melted paraffin, solidified and cut into thin slices. Paraffin sections were deparaffinized with xylene and alcohol, and then stained with hematoxylin and eosin dyes.

### Oxidative biomarkers measurement

Total reactive oxygen species (ROS)/NRS levels in the tissue homogenates were measured using the Cellular ROS Assay Kit (Cat. No. STA-347, Cell Biolabs, Inc. CA, USA). Levels of malondialdehyde (MDA), reduced GSH, as well as activity of MnSOD, were measured in the renal homogenates using commercial colorimetric assay kits (Cat. No. NWK-MDA01, NWLSS, USA, Cat. No. 7511–100-K, Trevigen, Gaithersburg, MD, USA, and Cat No. EIASODC, Thermo Fisher, USA; Cat. No. MAK190-1KT, Sigma Aldrich, UK), respectively. GSSG (Nanjing Jiancheng Company, Shanghai, China) and glutathione (GSH) (Beyotime, Shanghai, China) were evaluated by commercial kits. All samples were measured for *n* = 6/group and were performed in accordance with the manufacturer’s instructions [20].

### ELISA assay

The ELISA assay was performed to demonstrate the cytokine levels in the renal tissues according to the manufacturer’s protocol. The serum inflammatory factors including TNF-α (#BMS607-2INST), IL-6 (#BMS603–2), IL-10 (#BMS614INST) and MCP-1 (#BMS6005) were measured by commercially available kits (Life Technologies, Carlsbad, CA, USA) [20].

### Quantitative real-time PCR

The kidney tissue of each mouse was thoroughly ground and mixed with 1 mL Trizol (Life Technologies, Carlsbad, CA, USA) in a centrifuge tube. Chloroform and isopropanol (Life Technologies, Carlsbad, CA, USA) were used to extract RNA from tissues. FastKing cDNA reverse transcription kit (TIANGEN Biotech, Beijing, China) was used to remove genomic DNA from RNA and reverse transcribe RNA into cDNA. SuperReal PreMix Plus (TIANGEN Biotech, Beijing, China) kit was used to perform qRT-PCR, in which SYBR green (TIANGEN Biotech, Beijing, China) was used as the fluorescent signal. The mixture configured according to its instructions was placed in a Roche fluorescent quantitative PCR machine, and the LightCycler&reg480 software was used to analyze the data with built-in algorithm. *GAPDH* was used as a negative control. The primers used in this research were as follows:

- *IL-6*: primer F, 5′-ACAACCACGGCCTTCCCTACTT-3′,
- primer R, 5′-CACGATTTCCCAGAGAACATGTG-3′;
- *TNF-α* primer F, 5′-CTACCTTGTTGCCTCCTCTTT-3′,
- primer R, 5′-GAGCAGAGGTTCAGTGATGTAG-3′;
- *MCP-1* primer F, 5′- TAAAAACCTGGATCGGAACCAAA-3′,
- primer R, 5′- GCATTAGCTTCAGATTTACGGGT-3′.
- *IL-10* primer F, 5′-CTTACTGACTGGCATGAGGATCA-3′,
- primer R, 5′-GCAGCTCTAGGAGCATGTGG-3′.
- *GAPDH* primer F 5′-ACCCCAGCAAGGACACTGAGCAAG-3′,
- primer R 5′-GGCCCCTCCTGTTATTATGGGGGT-3′.

### Western blot

The mouse kidney tissue was thoroughly ground and mixed with 200 μl RIPA buffer. 200 μl of 2× loading buffer was added to the mixture. The mixture was placed in a metal bath at 100 °C to denature the protein. A 10% SDS-PAGE was utilized to separate the proteins in an electrophoresis apparatus with a constant voltage of 140 V for 120 min. The protein on SDS-PAGE was then transferred to a polyvinylidene difluoride membrane (Real-Times Biotechnology Co.,Ltd., Beijing, China) in a transfer tank with a constant current of 300 A. The membrane was co-incubated with anti-p-AMPK, anti-AMPK, anti-SIRT1 and anti-actin (1:1000, Cell Signaling Technology, Beijing, China) at 37 °C for 1 h and washed by phosphate buffered saline for 3 times. Then, the goat anti-rabbit secondary antibody (1:10000, Cell Signaling Technology, Beijing, China) was incubated with the membrane at 37 °C for 1 h. An AEC Peroxidase Substrate Kit (Solarbiotech, Shanghai, China) was used for blot imaging [21].

### Statistical analysis

The categorized variables were shown as frequency or percentage. One-way ANOVA test with a post hoc test was acquired, respectively, for the statistical analysis in this research. Mean ± SD was used to represent the data in our figures only. *P* < 0.05 were considered as the significant difference. GraphPad Prism 10.0 was used for the plotting and analysis of the data in this research.

## Results

### Effects of maslinic acid on the characteristics of diabetic mice

**Fig. 1:**
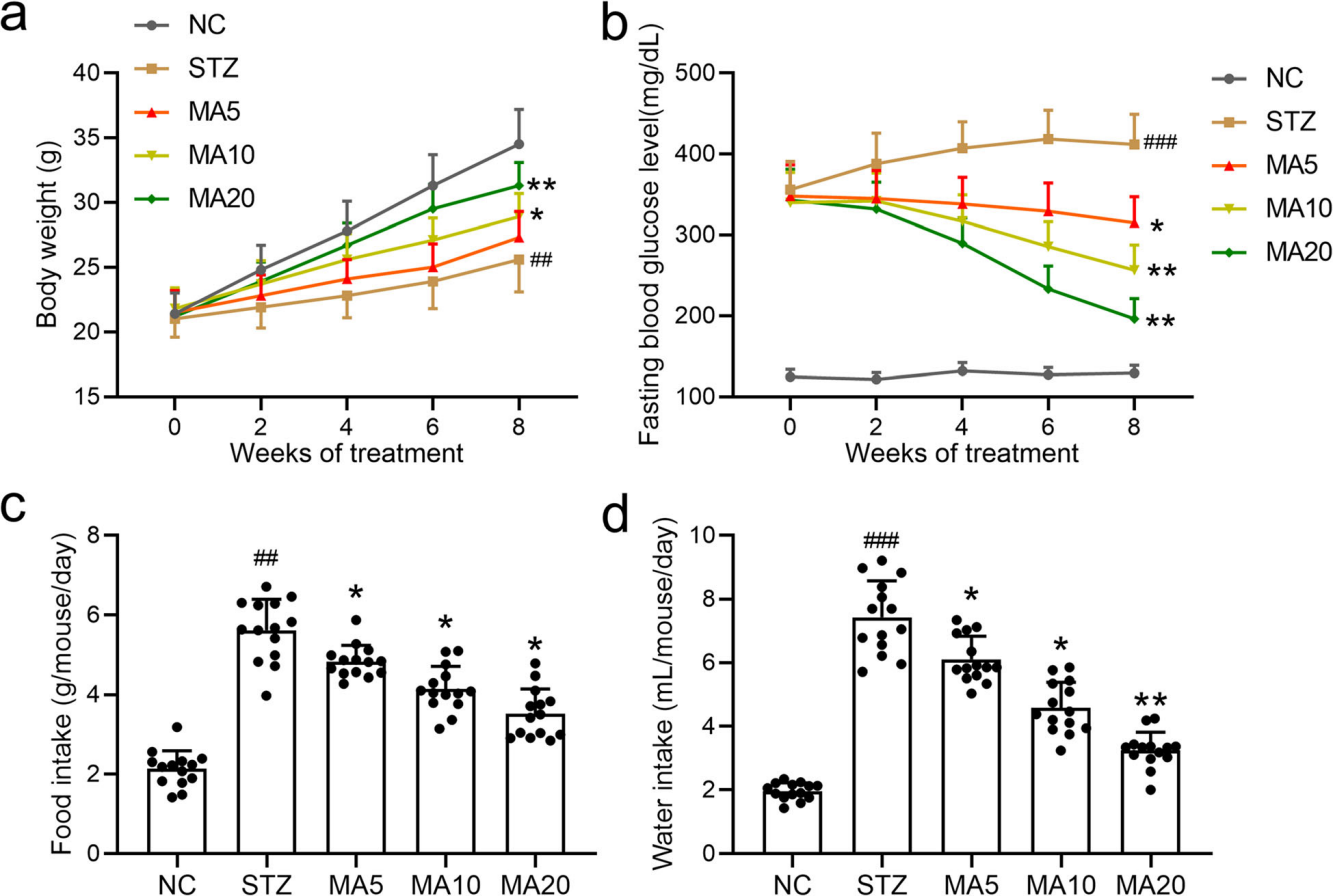
Effects of maslinic acid treatment on body weight (a), fasting blood glucose levels (b), average food intake (c) and average water intake (d) in mice model of diabetic nephropathy. All mice had free access to food and water at all times. The volume of consumed water and amount of food were recorded. Fasting blood glucose levels and body weight were measured once every 2 weeks for 8 weeks. *N* = 14 for each group. Data are presented as mean ± SD. ##*p* < 0.01, ###*p* < 0.001 compared to NC group, *p < 0.05, **p < 0.01compared to STZ group

To demonstrate the potential therapeutic effects of maslinic acid against diabetes, body weight, fasting blood glucose level, average food intake and average water intake were measured and analyzed. As shown in Fig. 1a, 20 mg/kg maslinic acid treatment significantly rescued the STZ-induced loss of body weight. Similarly, 5 mg/kg, 10 mg/kg and 20 mg/kg maslinic acid treatment markedly decreased the fasting blood glucose levels compared to the STZ group (Fig. 1b). In addition, 20 mg/kg maslinic acid significantly lowered the food and water intake in diabetic mice (Fig. 1c and d). Together, administration of different amounts of maslinic acid alleviated the symptoms of diabetic mice to varying degrees, and there were no significant side-effects of maslinic acid on diabetic mice. Accordingly, we chose 20 mg/kg as the dosage for long-term maslinic acid administration. There were no significant side effects of 20 mg/kg maslinic acid administration on average food intake (Fig. S1a), average water intake (Fig. S1b), kidney/body weight (Fig. S1c), urea (Fig. S1d), BUN (Fig. S1e) or creatinine (Fig. S1f) in the serum of normal group mice.

### Effects of maslinic acid on serum parameters in diabetic mice

**Fig. 2:**
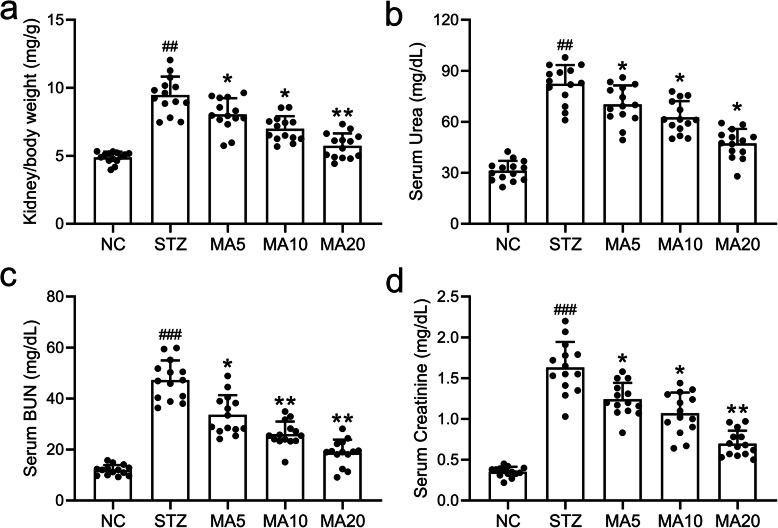
Effects of maslinic acid treatment on kidney/body weight (a), Urea (b), BUN (c) and Creatinine (d) in serum of mice model of diabetic nephropathy at the end of 8-week treatment. Serum levels of blood urea nitrogen (BUN), urea, and creatinine (Cr) were analyzed by an automatic analyzer (cobas® 8000 modular analyzer series. Roche Diagnostics). *N =* 14 for each group. Data are presented as mean ± SD. ##*p* < 0.01, ###*p* < 0.001 compared to NC group, *p < 0.05, **p < 0.01compared to STZ group

To investigate the effects of maslinic acid administration on renal functions, kidney/body weight, urea, BUN and creatinine in the serum of mice with diabetic nephropathy were measured by an automatic analyzer (*N* = 14 per group). As shown in Fig. 2a, STZ treatment induced renal fibrosis and increased kidney/body weight ratio. On the other hand, 20 mg/kg maslinic acid treatment significantly rescued the increased kidney/body weight ratio, suggesting that maslinic acid protected the nephron of mice with diabetic nephropathy from sclerosis. Similarly, 5 mg/kg, 10 mg/kg and 20 mg/kg maslinic acid treatment decreased serum urea, BUN and creatinine levels to varying degrees (Fig. 2b, c and d), suggesting that maslinic acid administration protected the renal function of mice against diabetic nephropathy.

### Effects of maslinic acid on urine parameters in diabetic mice

**Fig. 3:**
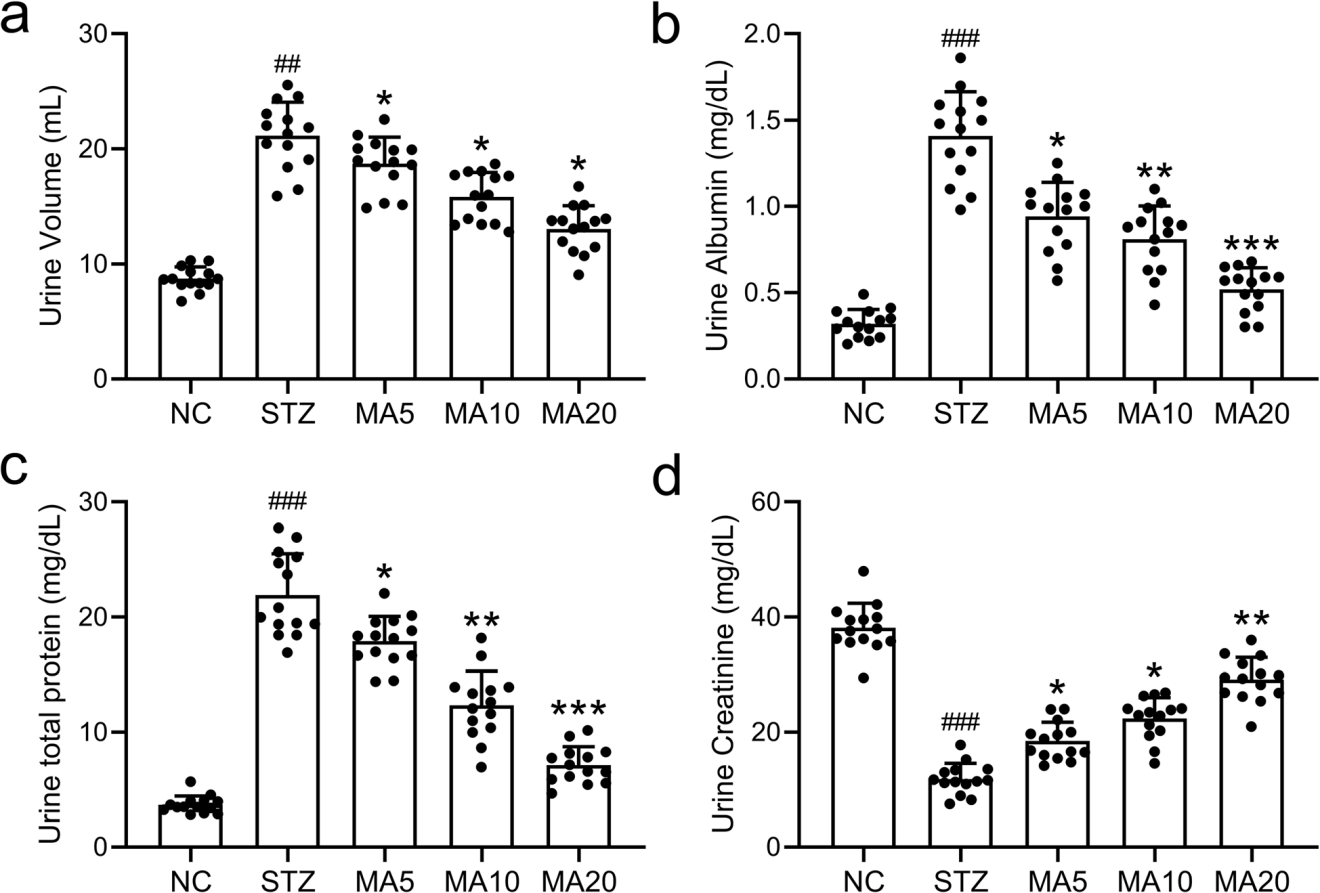
Effects of maslinic acid treatment on urine volume (a), urine albumin (b), total proteins (c) and Creatinine (d) in urine of mice model of diabetic nephropathy at the end of 8-week treatment. Urinary levels of albumin, total proteins, and creatinine were analyzed by an automatic analyzer (cobas® 8000 modular analyzer series. Roche Diagnostics). *N =* 14 for each group. Data are presented as mean ± SD. ##p < 0.01, ###*p* < 0.001 compared to NC group, *p < 0.05, **p < 0.01 and ***p < 0.001 compared to STZ group

To further demonstrate the nephroprotective role of maslinic acid against diabetic nephropathy, urine parameters including urine volume, urine albumin, total proteins and creatinine of diabetic mice after 8-week maslinic acid treatment were measured and analyzed, respectively. As shown in Fig. 3a, maslinic acid treatment alleviated the polyuria in diabetic mice induced by STZ. More importantly, maslinic acid treatment significantly lowered albumin and total proteins in the urine of diabetic mice, suggesting that maslinic acid protected the function of glomerulus against diabetic nephropathy (Fig. 3b and c). In addition, maslinic acid treatment also increased the creatinine in urine of mice with diabetic nephropathy (Fig. 3d), indicating that maslinic acid ameliorated the kidney filtration function.

### Effects of maslinic acid treatment on renal injury

**Fig. 4:**
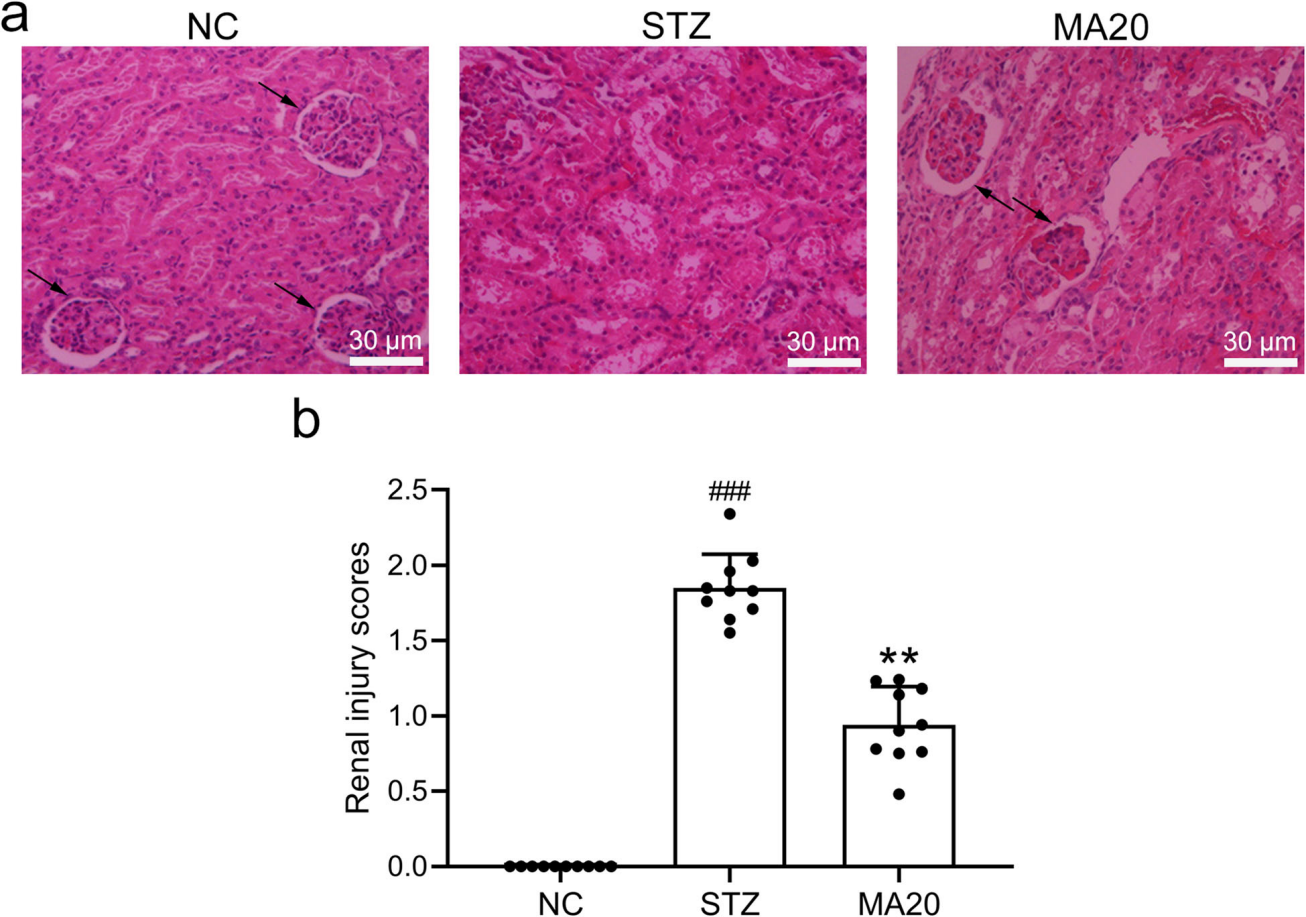
Effects of 20 mg/kg maslinic acid treatment for 8 weeks on renal injury in mice model of diabetic nephropathy. a) Representative image of renal tissue stained with hematoxylin and eosin (HE) and the quantification of renal injury score (b). *N* = 10 for each group. Data are presented as mean ± SD. ###p < 0.001 compared to NC group, **p < 0.01 compared to STZ group

As shown in Fig. 4a and b, STZ-induced mice showed upregulated mass of glomerulus epithelium, thicker Bowman’s capsule, enhanced vacuolization in both the proximal and distal tubules, and elevated arteriolopathy. On the other hand, 20 mg/kg maslinic acid treatment for 8 weeks protected the normal structures of glomeruli and tubules. 20 mg/kg maslinic acid treatment significantly ameliorated the renal injury induced by diabetes in mice. The damage quantification was graded using the following parameters: hemorrhage, inflammatory cell infiltration, tubular cell necrosis and apoptosis, cellular edema, and tubular dilatation based on a 4-score system (0 = histopathologic changes < 10%; 1 = 10 to 25%; 2 = 25 to 50%; 3 = 50 to 75%; and 4 = 75 to 100%)。A 4-point scoring range was used in this study, and damages less than 10% were considered as 0 points.

### Effects of maslinic acid treatment on renal oxidative stress

**Fig. 5:**
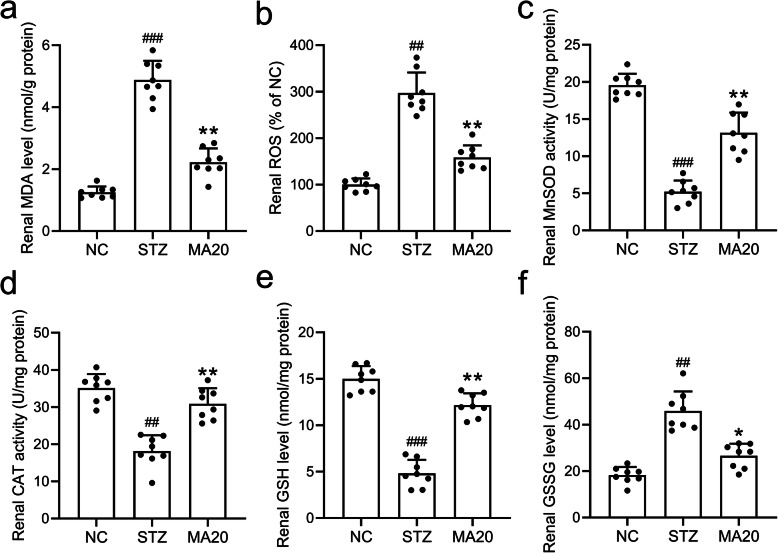
Effects of 20 mg/kg maslinic acid treatment for 8 weeks on renal oxidative stresses in mice model of diabetic nephropathy. The levels of MDA (a), ROS (b), MnSOD (c), CAT (d), GSH (e) and GSSG (f) in renal tissues were measured. *N* = 8 for each group. Data are presented as mean ± SD. ##p < 0.01, ###p < 0.001 compared to NC group, **p < 0.01 and ***p < 0.001 compared to STZ group

To further investigate the mechanisms for maslinic acid to inhibit the renal injury induced by STZ, the oxidative biomarkers including MDA, ROS, MnSOD, GSH and CAT were measured. As shown in Fig. 5a and b, 20 mg/kg maslinic acid treatment for 8 weeks down-regulated MDA and ROS levels in the renal tissues of diabetic mice, suggesting that maslinic acid decreased the oxidative stress in the kidney. Meanwhile, 20 mg/kg maslinic acid treatment upregulated the activities of enzymes targeting ROS, including MnSOD, CAT and GSH (Fig. 5c, d and e), while downregulated the GSSG activity (Fig. 5f). Thus, one of the possible mechanisms by which maslinic acid protected the renal tissues of diabetic mice was to reduce the oxidative stress in the kidney.

### Effects of maslinic acid treatment on renal inflammation

**Fig. 6:**
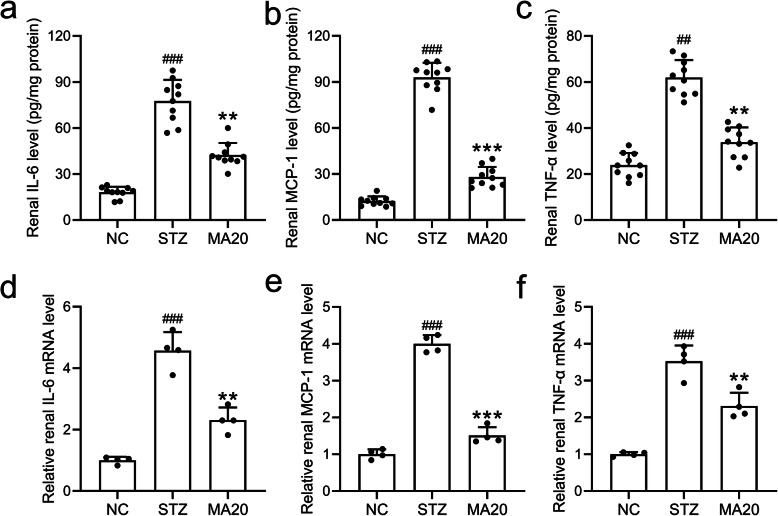
Effects of 20 mg/kg maslinic acid treatment for 8 weeks on renal inflammatory responses in mice model of diabetic nephropathy. Levels of IL-6 (a), MCP-1 (b) and TNF-α (c) in the renal tissues of diabetic nephropathy mice. mRNA levels of IL-6 (d), MCP-1 (e) and TNF-α (f) in the renal tissues were tested by qRT-PCR. Relative expression pattern was analyzed by comparative threshold cycle (2-ΔΔct) method and normalized to NC group. *N =* 10 for each group. Data are presented as mean ± SD. ##p < 0.01, ###p < 0.001 compared to NC group, **p < 0.01 and ***p < 0.001 compared to STZ group

Inflammatory factors including IL-6, MCP-1 and TNF-α in the renal tissue and serum of mice in different groups were evaluated by ELISA and qRT-PCR assays. The renal pro-inflammatory cytokines IL-6, MCP-1 and TNF-α increased with STZ induction, which were significantly lowered by 20 mg/kg maslinic acid treatment (Fig. 6a, b and c). Similarly, the circulating levels of pro-inflammatory cytokines including IL-6, MCP-1 and TNF-α were also decreased by the treatment of maslinic acid in diabetic mice (Fig. 6d, e and f). Moreover, 20 mg/kg maslinic acid treatment upregulated both the protein (Fig. S2a) and mRNA levels of IL-10 (Fig. S2b) in the renal tissues of diabetic nephropathy mice. These data suggested that maslinic acid inhibited inflammation during the progression of diabetic nephropathy.

### Maslinic acid treatment activated the renal AMPK/SIRT1 signaling pathway

**Fig. 7:**
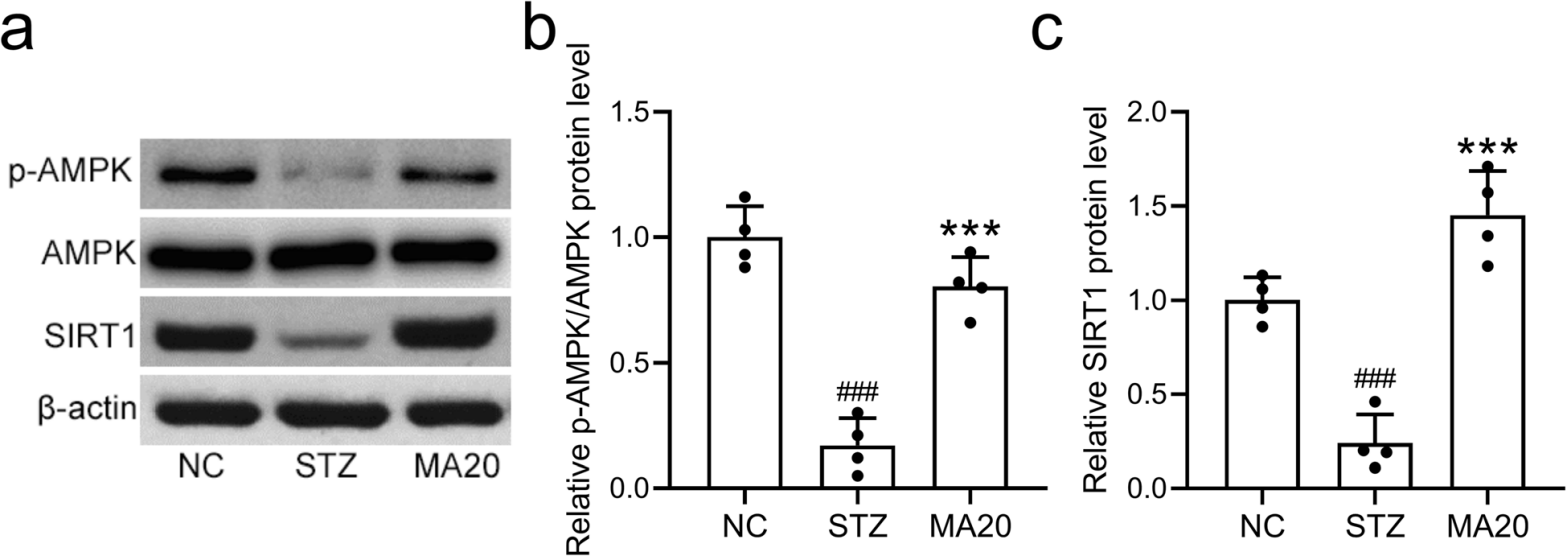
Maslinic acid treatment for 8 weeks activated renal AMPK/SIRT1 signaling pathway in mice model of diabetic nephropathy. Western blotting was used to measure the protein expressions of p-AMPK, AMPK and SIRT1 (a) and the relative expressions were normalized to NC. *N =* 10 for each group. Data are presented as mean ± SD. ###p < 0.001 compared to NC group, ***p < 0.001 compared to STZ group. A The western blotting assay was used to measure the protein expressions of p-AMPK, AMPK and SIRT1 in renal tissues of mice in STZ, MA20 and NC group. All the blots in the above figure were arranged from left to right in the order of NC, STZ, and MA20. The three blots on the left side of the upper half of the picture were p-AMPK, and the blots on right side were AMPK. The top three blots in the lower half of the picture were SIRT1, and the bottom three blots were beta-actin

To further demonstrate the molecular mechanisms underlying the nephropathy effect of maslinic acid, Western blot assay was performed. As shown in Fig. 7a and b, maslinic acid treatment induced the phosphorylation and activation of AMPK, which subsequently induced the upregulation of SIRT1 (Fig. 7a and c). Since AMPK and SIRT1 play crucial roles in the oxidative protection and anti-inflammatory activity, maslinic acid likely protected the renal function of diabetic mice via activating the AMPK/SIRT1 signaling pathway.

## Discussion

As of 2015, about 415 million people in the world were suffering from diabetes, and by 2040 this number is expected to increase to 642 million [22]. Diabetic nephropathy is currently one of the most common complications of diabetes [23]. The ESRD caused by diabetic nephropathy has a very high morbidity and mortality rate worldwide. Mogensen et al. divided the progression of diabetic nephropathy into five stages according to its disease course and pathophysiological process: stage 1, renal hyperfunction and hypertrophy, and increased urinary albumin excretion; stage 2, no obvious morphological damage and increased glomerular filtration rate (GFR); stage 3, initiation of diabetic nephropathy, continuous proteinuria, and increased albuminuria and GFR; stage 4, overt diabetic nephropathy, persistent proteinuria (> 0.5 g/24 h), decreased GFR caused by high blood pressure; stage 5, uremic end-stage renal failure due to diabetic nephropathy [24]. Thus, it is urgent to explore new therapeutic strategies to reverse the damaged renal function in stage 1 and 2 and alleviate the symptoms of diabetic nephropathy in stage 3 and 4.

The pathogenesis of diabetic nephropathy is mainly affected by several factors, including disordered glucose metabolism, altered renal blood flow, abnormal expression of cytokines, genetic factors, elevated blood pressure and abnormal blood lipid levels [25]. Among them, the glucose-metabolism disorder is a core and classic cause of diabetic nephropathy [26]. The kidney is an important metabolic site for advanced glycation end products (AGEs) [27]. Abnormal glucose metabolism in diabetic patients can cause the accumulation of AGEs that severely damage the kidneys [28]. The deposition of AGEs in the glomerular basement membrane, glomerular mesangial cells, endothelial cells and podocytes could alter the structure of the glomerular basement membrane, cause abnormal filtration membrane function, and significantly increase extracellular matrix, which ultimately lead to glomerular sclerosis and proteinuria [29, 30]. Thus, it is crucial for clinicians to identify agents with both nephroprotective effects and metabolic regulatory functions to treat diabetic nephropathy.

In this study, we reported that maslinic acid was a new agent for the treatment of diabetic nephropathy with potent effects. First of all, maslinic acid itself exhibited the effect of regulating blood glucose levels and improving the systemic metabolism of diabetic mice. The hypoglycemic effect of maslinic acid could reduce the deposition of AGEs in the kidneys of diabetic mice, thereby protecting kidney function [16]. Secondly, maslinic acid could further protect the kidney function of diabetic mice by reducing the oxidative stress and inflammation [14]. Compared with other traditional medicines, maslinic acid exhibits multiple protective effects on kidney function, making it more advantageous in the treatment of diabetic nephropathy [31].

Maslinic acid belongs to the class of triterpene acids and is a derivative of oleanolic acid. Maslinic acid can significantly inhibit the elevation of blood glucose level caused by adrenaline and glucose, as well as the degradation of liver glycogen caused by adrenaline [32]. Maslinic acid increases liver glycogen content in mice with glucose-induced hyperglycemia, as well as in normal mice without affecting normal blood sugar levels [33]. A previous research shows that maslinic acid has obvious anti-oxidant properties in cultured cells in vitro [34]. It has also been reported that maslinic acid inhibits the expression of arterial inducible nitric oxide ribozyme (iNOS) gene in the mouse peritoneal macrophages following treatment of lipopolysaccharide [35], suggesting the strong anti-inflammation effect of maslinic acid. Prostaglandins are lipid products generated from arachidonic acid by the action of cyclooxygenase (COX) enzymes, and their activity can be blocked by maslinic acid [36]. Maslinic acid can also regulate the inflammation pathways through modulating the arachidonic acid metabolism including the nuclear factor-kappa B (NF-κB)/COX-2 expression, upstream protein kinase signaling, and phospholipase A2 enzyme activity, to protect tissue functions [37]. Scientists at the University of Granada in Spain found that maslinic acid can inhibit the activation of serine proteases, by which HIV can release itself from the infected cells to the outside and spread to the body of the virus-infected individual [38]. Thus, maslinic acid can reduce the spread of HIV in the body by 80%. In addition to being widely recognized for its antioxidant, anti-inflammatory, antibacterial and antiviral properties, maslinic acid has also been reported to exhibit anti-diabetic effects by some studies. For instance, Hung et al. demonstrated that maslinic acid played an important role in the protection of cardiovascular systems in diabetic mice when administrated with asiatic acid [17]. In addition, maslinic acid has been reported to enhance the insulin signaling pathway and inhibit glycogen phosphorylase to regulate glycogen metabolism [39]. Similarly, we also reported the anti-diabetic effects of maslinic acid in this article. We showed that 20 mg/kg maslinic acid treatment effectively reduced the blood sugar content of diabetic mice for about 8 weeks. We also demonstrated that maslinic acid could suppress the pro-inflammatory factors while upregulate anti-inflammatory factor (IL-10) to inhibit renal inflammation. It also inhibited the oxidative stress in renal tissues, which was consistent with the results of previous studies [40, 41].

AMPK is an important energy sensor, which can sense the energy metabolism state in the body and regulate the energy metabolic process by altering the expression or activity of its downstream genes [42]. AMPK can transcriptionally activate nicotinamide phosphoribosyl transferase (Nampt), which increases the ratio of NAD+/NADH and activates another energy sensor, Sirt1. The activation of AMPK and Sirt1 strengthens the body’s catabolism and weakens the anabolism, thus reducing blood glucose levels and maintaining the metabolic homeostasis [19]. Insulin resistance plays an important role in the pathogenesis of diabetic nephropathy. In insulin-resistant organs, AMPK pathway activation can increase insulin sensitivity. The specific mechanism is related to the inhibition of mTOR/p70S6K pathway [43]. In this study, we demonstrated for the first time that the treatment of maslinic acid can effectively promote the phosphorylation of AMPK in the kidney tissue of diabetic mice. The activation of AMPK further up-regulated the expression of SIRT1. The activation of AMPK/SIRT1 signaling pathway may affect the metabolism in the kidney of diabetic mice, thereby protecting the renal function and alleviating the symptoms of diabetic nephropathy.

## Conclusions

In conclusion, we reported that maslinic acid administration alleviated the diabetic nephropathy in mouse model. Maslinic acid treatment not only reduced the blood sugar level of diabetic mice, but also inhibited the oxidative stress and inflammation in their kidneys. Therefore, maslinic acid treatment can protect the structure and function of the kidney in diabetic mice. We believe that our research could provide new evidence to support the clinical application of maslinic acid.

## Supplementary Information

**Additional file 1.**

## Data Availability

The datasets supporting the results of this article are included within the article. Further enquiries can be directed to the corresponding author.

## Abbreviations

ESRD: end-stage renal disease
AMP: Adenosine 5′-monophosphate
AMPK: activated protein kinase
STZ: Streptozocin
MA5: diabetic mice treated with 5 mg/kg maslinic acid
MA10: diabetic mice treated with 10 mg/kg maslinic acid
MA20: diabetic mice treated with 20 mg/kg maslinic acid
BUN: blood urea nitrogen
MDA: malondialdehyde
ROS: Reactive oxygen species
MnSOD: Manganese superoxide dismutase
GSH: glutathione
GFR: glomerular filtration rate
AGEs: advanced glycation end products
iNOS: inducible nitric oxide ribozyme
