# Supplementary material for: Maslinic acid activates renal AMPK/SIRT1 signaling pathway and protects against diabetic nephropathy in mice
Source: BMC Endocr Disord. 2022 Jan 18;22:25. doi: 10.1186/s12902-022-00935-6 (PMC8767743; doi:10.1186/s12902-022-00935-6)
Supplement: Supplementary file 1 — Additional file 1. [file 12902_2022_935_MOESM1_ESM.docx]

Supplementary materials


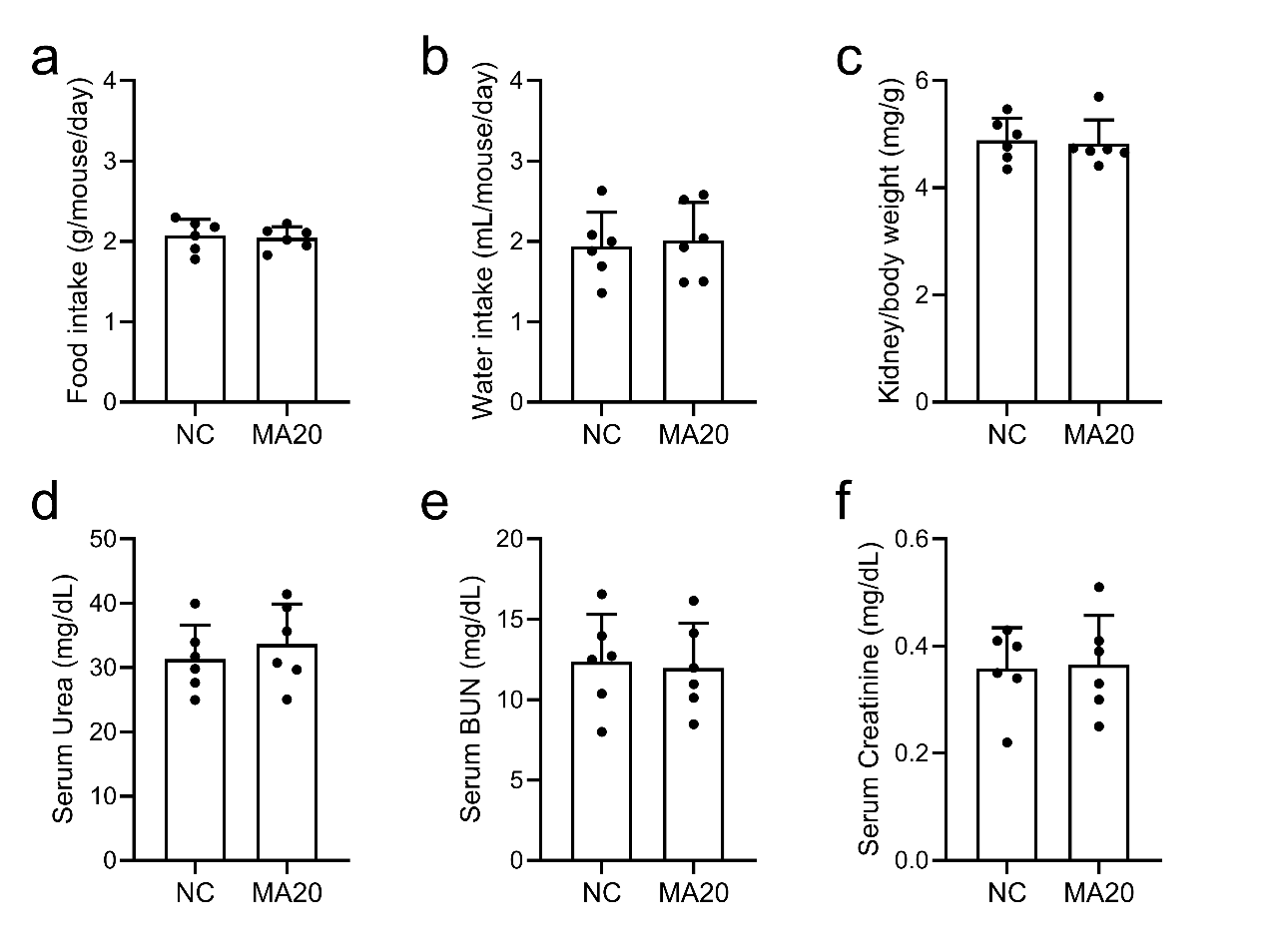


Figure S1. Effects of 20 mg/kg maslinic acid treatment for 8 weeks on the control mice. average food intake (a), average water intake (b), kidney/body weight (c), Urea (d), BUN (e) and Creatinine (f) in serum of mice were compared. There were no significant difference between the two groups.


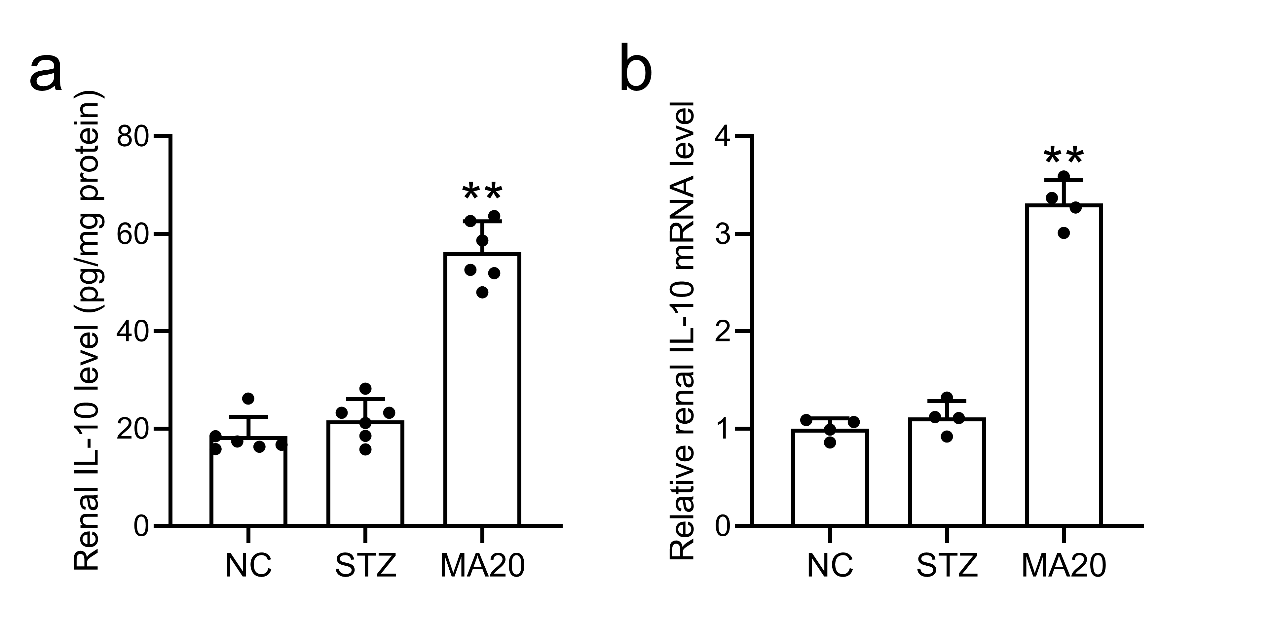


Figure S2. Effects of 20 mg/kg maslinic acid treatment for 8 weeks on renal anti-inflammatory responses in mice model of diabetic nephropathy. Levels of IL-10 (a) in the renal tissues of diabetic nephropathy mice. mRNA levels of IL-10 (b) in the renal tissues were tested by qRT-PCR. Relative expression pattern was analyzed by comparative threshold cycle (2-ΔΔct) method and normalized to NC group. Data are presented as mean ± SD. **p < 0.01 compared to STZ group.
